# Generation of Blue Electro-Optic Frequency Combs via a Four-Wave-Mixing Process in Rubidium Atomic Vapor

**DOI:** 10.1021/acsphotonics.6c00470

**Published:** 2026-08-04

**Authors:** Roy Zektzer, Ashish Chanana, Khoi Tuan Hoang, Rahul Shrestha, David A. Long, Kartik Srinivasan

**Affiliations:** † Joint Quantum Institute, NIST/University of Maryland, College Park, Maryland 20742, United States; ‡ Microsystems and Nanotechnology Division, 10833 National Institute of Standards and Technology, Gaithersburg, Maryland 20899, United States; § Faculty of Engineering, 26731 Bar-Ilan University, Ramat-Gan 5290002, Israel

**Keywords:** electro-optic frequency combs, four-wave mixing, rubidium vapor, frequency conversion, blue light generation, atomic physics, nonlinear optics

## Abstract

Electro-optic frequency combs (EO combs) enable high spectral and temporal resolution in atomic, molecular, and cavity-based spectroscopy that is at the heart of many quantum sensing applications. However, producing EO combs in the visible range remains challenging due to the limited availability of key components such as narrow-linewidth lasers and electro-optic phase modulators. Rubidium (Rb) is a highly nonlinear material that supports various four-wave mixing (4WM) processes, which are utilized for spectral translation and the generation of squeezed light. Here, we show the spectral translation of a 780 nm EO comb to 420 nm (a spectral separation of 330 THz) via a 4WM process in Rb vapor. We demonstrate the capability to extend the output comb bandwidth and signal-to-noise ratio by varying input power levels. Through this coherent process, we can generate frequency-agile combs in the blue, including a 1 kHz spaced comb with over one million individual comb teeth, of relevance for spectroscopic measurement of narrow-linewidth features such as electromagnetically induced transparency. Leveraging this approach across different 4WM interactions in Rb should allow for further wavelength conversion and the generation of correlated frequency combs, paving the way for sub-shot-noise (squeezed-light) measurements.

## Graphical abstract

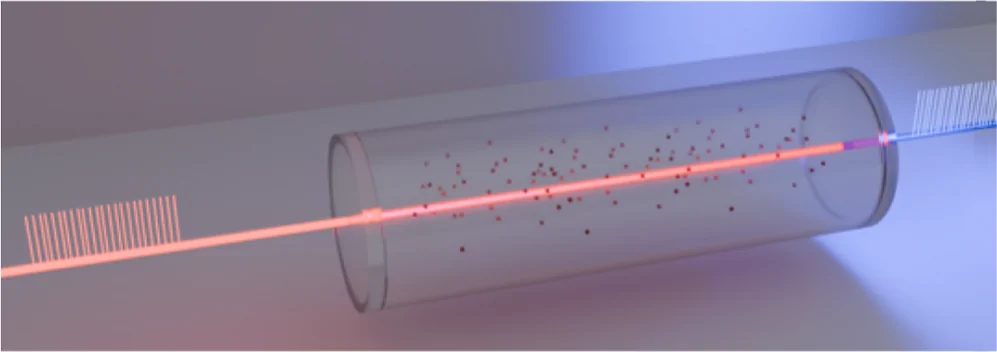

## Introduction

Optical frequency combs have revolutionized the field of metrology by providing a coherent bridge between optical and microwave frequencies,^1,2^ as well as enabling advancements in areas such as optical atomic clocks,^3^ laser ranging,^4^ and spectroscopy.^5^ For sensing and spectroscopy applications, electro-optic (EO) combs generated by modulating continuous-wave lasers^6,7^ are particularly appealing, as they enable frequency-agile, digitally controlled repetition rates and bandwidths. This allows for custom spectral and temporal resolutions to suit the specific requirements of selected materials and applications. For instance, EO combs have been employed in molecular spectroscopy across spectral regions ranging from the ultraviolet to the mid-infrared,^6,7^ enabling time resolution as fast as 20 ns.^8^ The mutual coherence of the comb teeth allows for measurement of sub-kHz spectroscopic features such as those encountered in atomic physics,^9^ enabling quantum applications, as shown in recent demonstrations with Rydberg electrometry.^10–12^

Given their broad range of applications, there is a strong demand for EO combs across a wide wavelength range with sufficient power to achieve high signal-to-noise ratio (SNR) measurements. However, generating EO combs far outside the telecommunications (telecom) bands presents a major challenge. These spectral regions include some of the most scientifically valuable targets for spectroscopy, yet EO technologies at these wavelengths are still relatively underdeveloped. Spectral translation of EO combs^8,10,13,14^ from more technologically mature wavelengths to various other wavelengths can solve this issue.

Atoms serve not only as a primary motivator for developing EO comb sources across visible, near-infrared, and mid-infrared wavelengths for applications such as radiofrequency sensing,^10^ frequency referencing,^15^ and magnetometry,^16^ but also can act as sources for these essential wavelengths through four-wave mixing (4WM) processes. Although limited in spectral bandwidth, atomic vapors exhibit significantly higher nonlinearity than conventional transparent materials.^17^ This enhanced nonlinearity has been leveraged for numerous applications, including the generation of squeezed light,^18^ the transduction of single photons from the near-infrared to telecom wavelengths,^19^ and the conversion of near-infrared photons to mid-infrared photons.^20^

In resonantly driven atomic vapors, nonlinear optical processes arise from the coherent interaction between multiple optical fields and the atomic dipole polarization. In particular, four-wave mixing processes can generate new optical frequencies through atomic coherence between ladder or cascade-connected states, enabling efficient spectral translation between widely separated wavelengths. Unlike conventional nonlinear crystals, where phase matching is primarily determined by material dispersion, atomic systems can exploit discrete resonances and long-lived coherences to satisfy energy and momentum conservation while strongly enhancing the effective third-order susceptibility χ^(3)^. Moreover, because atomic vapors can support energy level structures in which states are connected by multiple transitions that can be widely varying in frequency, they provide a natural mechanism for frequency conversion across large spectral gaps that can present phase-matching challenges for conventional nonlinear media. These properties have enabled processes such as the generation of coherent blue light from near-infrared excitation in rubidium vapors and photon translation from 780 nm to telecom^21,22^

Here, we utilized a 4WM process in rubidium (Rb) to spectrally translate a 780 nm electro-optic (EO) comb to 420 nm (a spectral separation of 330 THz) and measured the output comb’s power and bandwidth as functions of the 780 and 776 nm input powers. Our results demonstrate the translation of frequency-agile combs, including a high-resolution, 1 kHz-spaced comb with over one million individual comb teeth. That generated blue comb spans nearly 2 GHz in width, making it well-suited for spectroscopy of both Doppler-broadened and sub-Doppler atomic features.

## Blue Electro-Optic Comb Generation

The Rb energy levels involved in our 4WM process are shown in Figure 1a. Through a ladder configuration involving 780 and 776 nm photons, atoms are excited to the 5*D*_5/2_ level and subsequently decay back to the ground level through the 6P_5/2_ level while emitting photons in the blue at 420 nm and in the mid-infrared at 5230 nm. This process typically results in fluorescence that is nearly isotropic. However, when momentum and energy conditions are precisely matched, it can generate coherent, directional light in the visible and mid-infrared spectral regions.^20^

**Figure 1:**
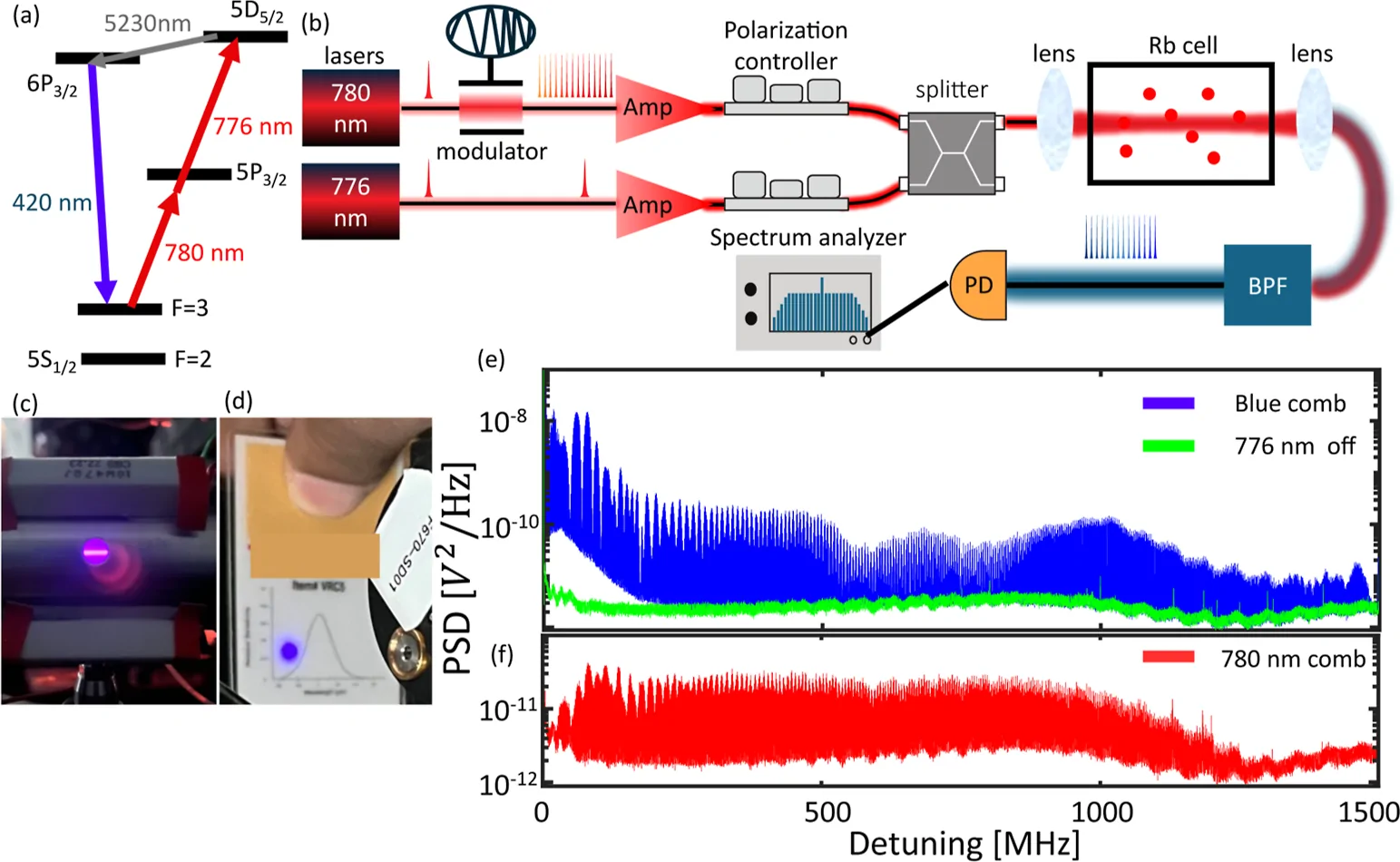
Concept for blue EO comb generation. (a) Energy level diagram of the four-wave mixing (4WM) process in rubidium (Rb). (b) Setup schematic: A 780 nm tunable laser is phase-modulated to generate an EO comb, which is then amplified. A 776 nm tunable laser is amplified as well, and both lasers’ polarizations are tuned by polarization controllers and combined using a fiber splitter. The combined output of the two lasers is collimated out of the fiber with a diameter of ≈1 mm and focused to a diameter of 50 μm into the center of a 5 cm long cell containing both ^85^Rb and ^87^Rb (natural abundance) at 100 °C. The cell’s output light is filtered using an optical bandpass filter (BPF) and measured using a fast photodetector (PD). The detector output is analyzed using a spectrum analyzer. (c) Image of the blue fluorescence from the side of the Rb cell. (d) Image of the coherent blue light generated via 4WM and observed at the output of the Rb cell. (e) Measured power spectral density (PSD) of the blue EO comb (blue) and the output when the 776 nm pump laser is turned off (green), with the latter demonstrating the lack of a blue EO comb in the absence of the pump light. (f) Corresponding PSD for the 780 nm input comb (red).

Our experimental setup is depicted in Figure 1b–d. We directed a 780 nm laser through an EO phase modulator driven by a frequency-chirped waveform of the form

$$h\left(t\right)=A\;\sin\left(2\pi \left(f_{0}t+\frac{\left(f_{1}-f_{0}\right)t^{2}}{2\Delta t}+\phi \right)\right)$$

where *A* is a constant amplitude, *f*_0_ and *f*_1_ are the chirp’s initial and final frequencies, respectively, Δ*t* is the chirp’s time duration, and ϕ is a constant phase term. Driving an electro-optic phase modulator with this chirped waveform results in a frequency comb with a tooth spacing determined by the inverse of the chirp duration (i.e., $\frac{1}{\Delta t}$ ) and a bandwidth equal to twice the chirp’s frequency span (i.e., 2­(*f*_1_ − *f*_0_))^23^ (see Figure 1f). The frequency chirp was produced by a commercial direct digital synthesis chip.^9^ When the amplitude, *A*, matches the half wave voltage (i.e., *V*_π_), the comb power is evenly divided such that one-third is distributed among the negative-order comb teeth, one-third among the positive-order comb teeth, and one-third is allocated to the carrier tone.

The comb output was amplified using a semiconductor optical amplifier, while the 776 nm laser was amplified with a tapered amplifier. We applied ≈16 mW of 780 nm power and ≈105 mW of 776 nm power for the results shown in Figure 1e. The polarizations of both lasers were adjusted using fiber polarization controllers before being combined in a fiber splitter and focused into a 5 cm long vapor cell heated to 100 °C and filled with natural abundance Rb comprised of 85 and 87 atomic mass isotopes. The beam exiting the cell was collimated and subsequently filtered with an optical bandpass filter to remove the near-infrared wavelengths. We used a 1 GHz bandwidth (3 dB) photodetector with a gain of 700 V/A to record the 4WM-generated comb. The output of this photodetector was amplified by approximately 14 dB using a 4 GHz RF amplifier before being recorded with a 3 GSa/s digitizer.

After tuning the 780 nm laser wavelength to the *F* = 3 ground level and the 776 nm frequency to resonance, blue fluorescence was observed emerging from the side of the cell (see Figure 1c). Next, we adjusted the lasers’ wavelengths until we detected the directional emission characteristic of the 4WM process, with a distinct blue spot appearing at the output of the cell (see Figure 1d). With additional optimization of the lasers’ wavelengths and polarizations, we reached ≈10 μW of blue power. Accounting for the fact that approximately one-third of the power is placed in the comb’s carrier as well as the large overall number of comb teeth, we can estimate the typical single tooth power to be ≈ 2 nW. At this point, the 780 nm laser was blue detuned by 1.3 GHz from *F* = 3 ground state, and the 776 nm laser was detuned by hundreds of MHz. The detuning of the 776 nm laser was roughly estimated by the piezo voltage detuning of the laser from the resonance transition.

Importantly, when a frequency chirp was placed on the EO modulator to produce a 3000 MHz wide frequency comb at 780 nm, we observed a corresponding frequency comb on the resulting blue light (see Figure 1e, blue data). As the comb consists of a strong carrier, we are measuring the beat note between the carrier and the comb teeth, and the presented data contains both positive- and negative-order comb teeth, resulting in a maximal measured bandwidth of 1.5 GHz. The bandwidth of this 4WM process, and hence the generated blue EO comb, might be expected to be limited due to the atomic transitions’ narrow linewidth; however we observe a >2000 MHz wide comb. This width is narrower than the 3000 MHz input comb due to both the photodetector’s 1000 MHz bandwidth (at the 3 dB point), as well as the 4WM process (see Figure 2a,b). We note this is much wider than the ≈400 kHz bandwidth resulting from the 6P_3/2_ lifetime. We attribute this increased bandwidth to power-broadening processes, Doppler broadening and the contribution from the two Rb isotopes (i.e., ^85^Rb and ^87^Rb). In order to verify we are not observing any leakage of the 780 nm pump comb, we turned off the 776 nm pump laser and confirmed we did not observe any comb structure (see Figure 1e, green data).

**Figure 2:**
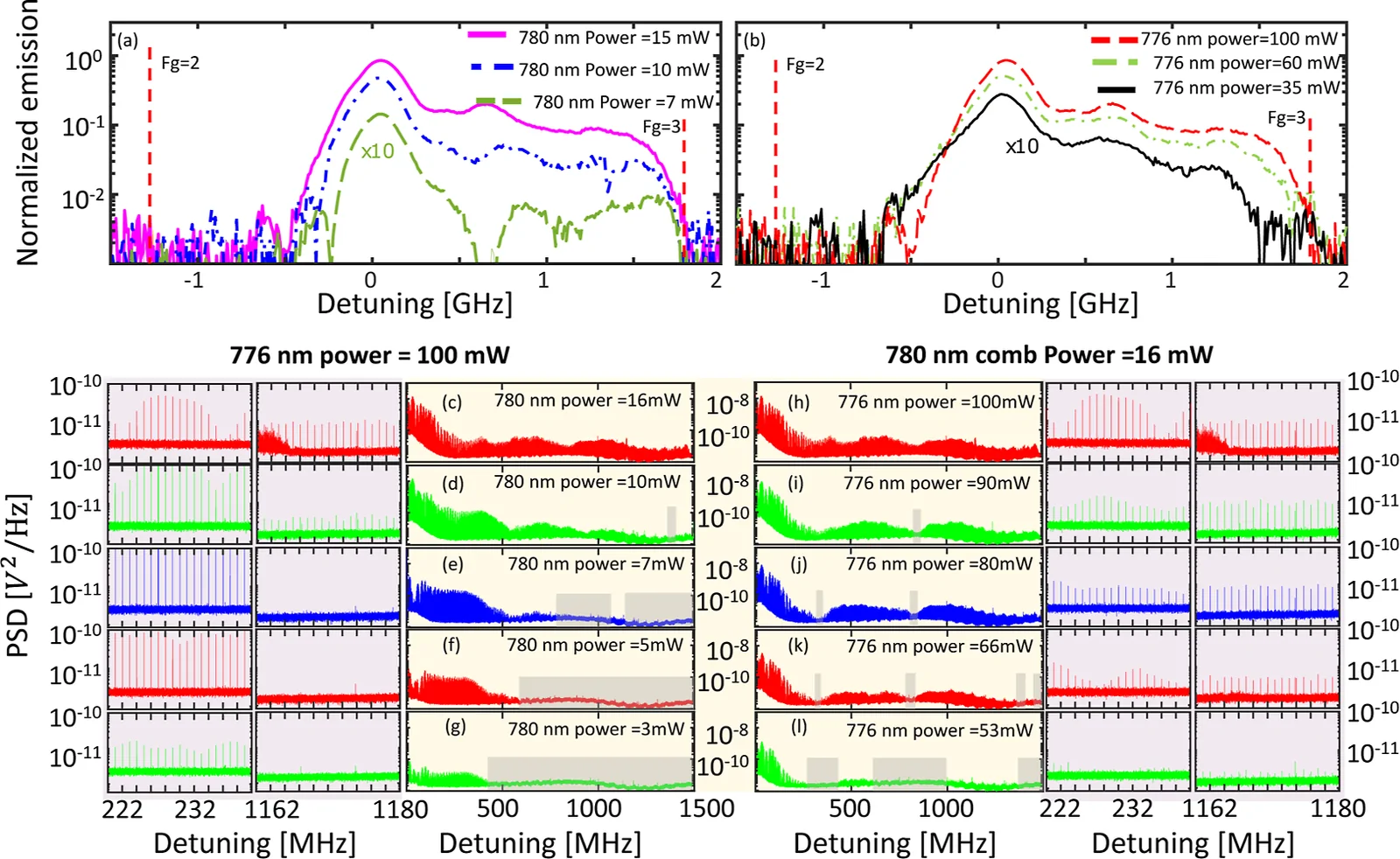
Dependence of the blue EO comb on applied optical power levels. (a)-(b) Spectra of generated coherent blue light at 420 nm with CW 780 and 776 nm lasers (i.e., without the EO comb). In (a), the 780 nm laser detuning and power are varied, and a fixed 776 nm power of 105 mW is used. In (b), the 780 nm detuning is varied for different 776 nm laser powers, and a fixed 780 nm laser power of 15 mW is used. In the (a) and (b) panels, the spectrum of the lowest power measurement is multiplied by *a* factor of 10 for visibility. The highest power represents ≈10 μW of coherent blue light. ^85^Rb transitions are marked with red dashed lines, with Fg being the associated F number of the ground level. In (c-l) we present the full comb bandwidth in the middle and focus on two spectral regions on the side of the image in order to clarify the bandwidth dependence on input powers. (c-g) Power spectral density (PSD) of the blue EO comb for different 780 nm comb power levels and a fixed 776 nm laser power of 100 mW. (h-l) PSD of the blue EO comb for different 776 nm pump power levels and a fixed 780 nm comb power of 16 mW. In (c-l), we mark in the gray squares the regions where the SNR is insufficient to detect comb teeth.

### Dependence of the Blue EO Comb on Optical Power and Rb Cell Properties

When comparing the 780 nm pump comb (Figure 1f) to the corresponding blue comb (Figure 1e), we observe that the pump comb appears significantly flatter. This difference arises because the blue EO comb’s shape, bandwidth, and total power are governed by the 4WM process in Rb, which is sensitive to the input laser powers, the length of the Rb vapor cell, and the atomic density.

To examine the blue comb’s dependency on the input powers of the 780 and 776 nm lasers, we conducted measurements under varying power conditions and compared them to continuous-wave (CW) spectroscopy obtained by scanning the 780 nm laser (see Figure 2). By examining the CW spectroscopy (see Figure 2a,b)), we see that increasing the 776 nm power enhances the total blue emission without significantly altering its spectral shape. The observed width of ≈2 GHz is in good agreement with our observed 4WM-generated comb found in Figure 1e. In contrast, varying the 780 nm power changes both the intensity and shape of the emission. This is because the 780 nm transition experiences much greater absorption, and is therefore, more susceptible to saturation effects along the propagation path,^24^ making its power profile more sensitive to input level. The resulting additional spectral lobes, previously observed and linked to detuning of the 776 nm laser,^20,25^ shift as the 780 nm power modifies the 5S_3/2_ population, thereby altering the 4WM conditions. Meanwhile, the 776 nm transition is more difficult to saturate and shows less sensitivity to input power.

Similar behavior is seen when using an EO comb as the 780 nm input. In Figure 2c–g, center, where the 780 nm power is varied (with 776 nm held constant), changes are observed in both the comb bandwidth and spectral shape. In contrast, varying the 776 nm power (Figure 2h–l), center) (with the 780 nm power fixed) primarily affects the overall signal strength, with little change in shape. For example, halving the 780 nm power leads to a noticeable drop in signal beyond 1 GHz detuning; while regions near 200 MHz maintain high SNR, those near 1170 MHz fall below the noise floor. This illustrates the bandwidth narrowing that occurs as the 780 nm power is reduced.

In contrast, reducing the 776 nm power results in a more uniform reduction in SNR across the comb spectrum. Notably, a 6 mW reduction in 780 nm power (i.e., by ≈ one-third) causes the comb signal around 1000 MHz to drop by over an order of magnitude, while a similar fractional change in 776 nm power (100 mW to 66 mW) produces a much smaller effect. Moreover, adjusting the 780 nm power shifts the positions of the secondary lobes and alters the comb shape, whereas changes to the 776 nm power do not. Interestingly, reducing the 780 nm power to 7 mW enhances the SNR near 200 MHz, suggesting power-dependent shaping of the emission spectrum. In our experimental setup, we could not increase the power any further due to damage limitations. Further increase of power will produce larger Rabi frequencies and increased bandwidth, but will eventually be limited by the Doppler shift and reduced atomic population at larger detunings.^26^

To further examine these effects, we have repeated this measurement with a shorter Rb cell (2 cm long), which allowed us to reach higher temperatures (up to 120 °C) with the same power source and hence higher vapor density. This cell, unlike the previous cell, contained only the ^85^Rb isotope. The resulting blue EO combs can be seen in Figure 3. While we can still observe the effect of the input comb power on the overall power of the blue EO comb, it seems to contain only one main lobe and does not have the several peaks we previously observed. This is consistent with the results shown in,^25^ which attributed this effect to Kerr lensing of the 780 nm beam at high vapor densities. The presence of only the ^85^Rb isotope likely also contributes to the observation of a smoother comb. Additionally, having a shorter cell also produces a more homogeneous power, leading to fewer variations due to power broadening and light shifts that can result in additional lobes in the 4WM process.

**Figure 3:**
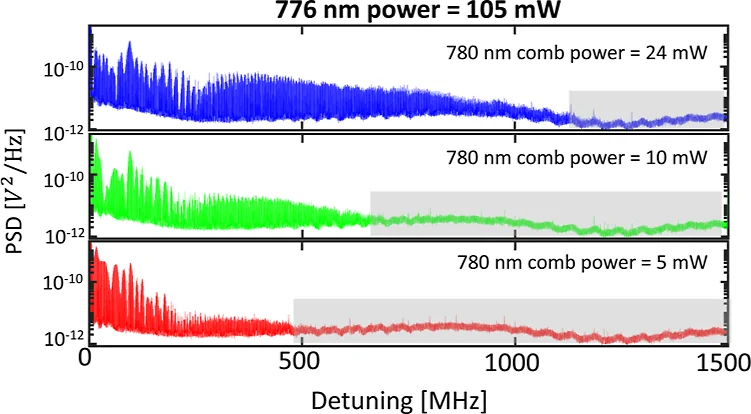
Power spectral densities (PSDs) of the blue EO comb when using a ^85^Rb cell of length 2 cm at 120 °C. (a–c) PSDs of the blue EO comb for different 780 nm comb powers. In each case, the pump comb was 1500 MHz wide with a 1 MHz spacing. We mark in gray squares regions where the SNR is insufficient to detect a comb tooth.

### Frequency Agility

Importantly, the generated blue EO combs preserve the frequency-agile nature of the initial near-infrared combs. As can be seen in Figure 4 we can readily produce 420 nm combs with spacings of 100 kHz, 10 kHz, and 1 kHz. In the case of the 1 kHz spaced comb, this corresponds to the spectral translation of more than 1 million individual comb teeth. These high-resolution combs could be used for the spectroscopy of narrow-linewidth features such as the sub-Doppler features of the 420 nm transition^27^ or electromagnetically induced transparency (EIT). While the power per comb tooth decreases as we increase the total number of comb teeth, the blue EO comb could be amplified by a variety of methods, including injection locking of a Fabry–Perot laser to the comb.^14^

**Figure 4:**
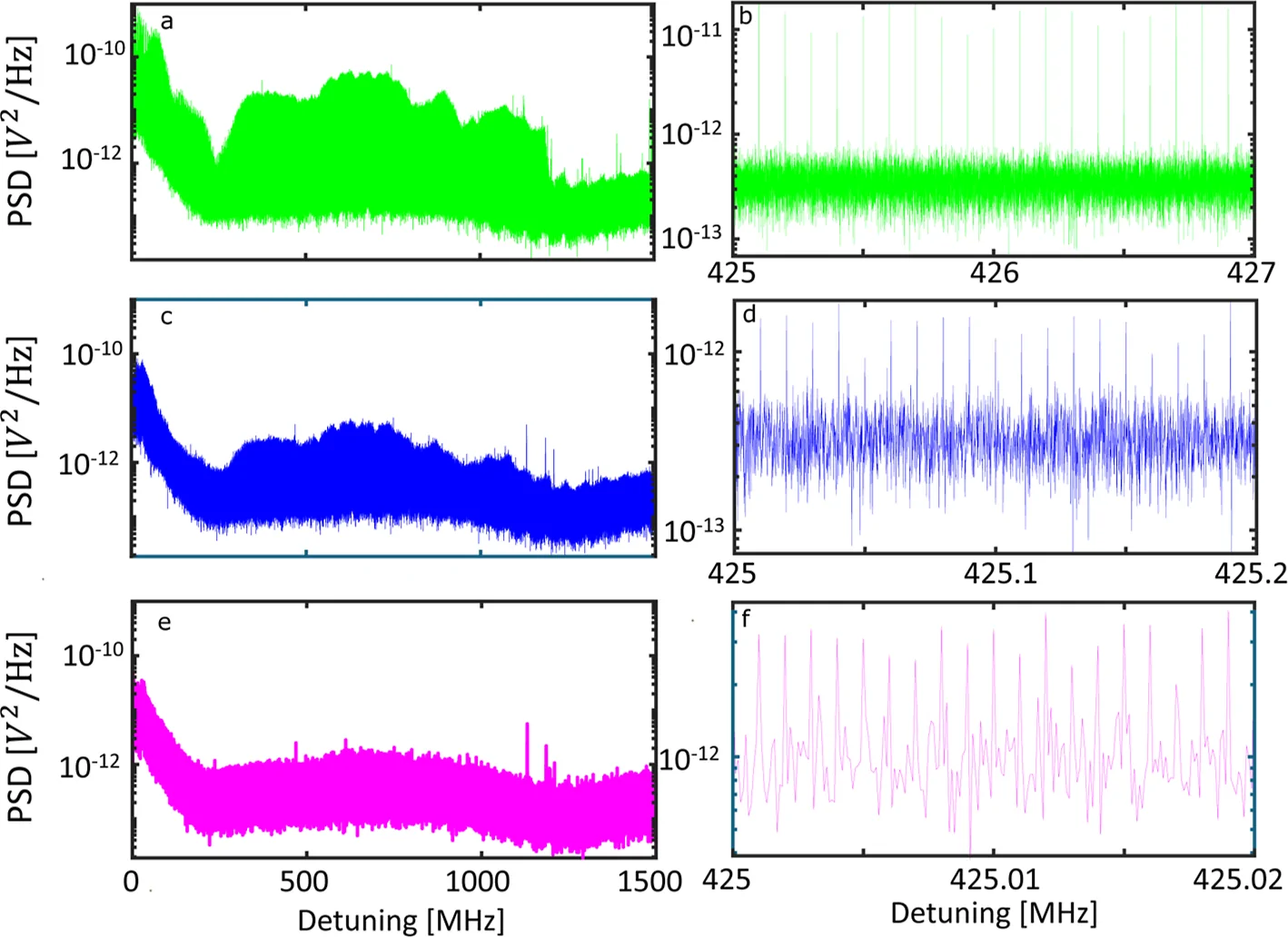
Blue EO comb frequency agility (a) Power spectral density (PSD) for a 1500 MHz wide blue EO comb with 100 kHz spacing. (b) Zoom in on 20 teeth of this 100 kHz spaced comb. (c) PSD for a 1500 MHz wide blue EO comb with 10 kHz spacing. (d) Zoom in on 20 teeth of this 10 kHz spaced comb. (e) PSD for a 1500 MHz wide blue EO comb with 1 kHz spacing. (f) Zoom in on 20 teeth of this 1 kHz spaced comb.

## Conclusion

In summary, we have shown the spectral translation of a high-resolution 780 nm comb to 420 nm using a 4WM process in Rb. We have shown that the bandwidth and power of the 420 nm comb are highly dependent on the input powers and the atomic density. We note that there are existing methods to improve the efficiency of the process, as was shown in ref.,^25^ where this 4WM process in Rb produced 1.1 mW output power with tens of mW of input power. While we are already able to measure high-SNR frequency combs spanning 2 GHz, such methods could enhance the signal by up to 2 orders of magnitude and substantially improve the SNR in Doppler-broadened measurements. A frequency-converted local oscillator with ≈1 mW of power will also enable saturation spectroscopy.^27^ Probing Rydberg transitions at 420 nm^28^ requires single comb tooth powers of several microwatts, which can require tens of milliwatts of total comb power. To realize such high-power combs we can effectively amplify the comb output after the 4WM process by injection locking a high-power Fabry–Perot laser diode to our comb.^14^ Future work will aim to improve the efficiency of this 4WM process by focusing the light to submicron features using nanophotonic waveguides.^29–33^ Further, while we were unable to measure it due to the window materials of our cell, a mid-infrared 5230 nm comb is also generated in this process.^20^ This wavelength regime also suffers from a lack of modulators and narrow line width lasers, as a result this comb could likewise see spectroscopic applications. Finally, the concepts of this measurement can be applied to other 4WM processes in Rb for applications such as squeezed light generation,^18^ allowing for applications including quantum^34^ and beyond-shot-noise-limit sensing.^35^
